# Inhibition of lateral shoot formation by RNA interference and chemically induced mutations to genes expressed in the axillary meristem of *Nicotiana tabacum* L.

**DOI:** 10.1186/s12870-021-03008-3

**Published:** 2021-05-27

**Authors:** Kaori Hamano, Seiki Sato, Masao Arai, Yuta Negishi, Takashi Nakamura, Tomoyuki Komatsu, Tsuyoshi Naragino, Shoichi Suzuki

**Affiliations:** Leaf Tobacco Research Center, Japan Tobacco Inc., 1900 Idei, Oyama, Tochigi 323-0808 Japan

**Keywords:** Chemically induced mutation, Lateral shoot inhibition, RNAi, RNA-seq, Sucker, Tobacco

## Abstract

**Background:**

Lateral branches vigorously proliferate in tobacco after the topping of the inflorescence portions of stems for the maturation of the leaves to be harvested. Therefore, tobacco varieties with inhibited lateral shoot formation are highly desired by tobacco farmers.

**Results:**

Genetic inhibition of lateral shoot formation was attempted in tobacco. Two groups of genes were examined by RNA interference. The first group comprised homologs of the genes mediating lateral shoot formation in other plants, whereas the second group included genes highly expressed in axillary bud primordial stages. Although “primary” lateral shoots that grew after the plants were topped off when flower buds emerged were unaffected, the growth of “secondary” lateral shoots, which were detected on the abaxial side of the primary lateral shoot base, was significantly suppressed in the knock-down lines of *NtLs*, *NtBl1*, *NtREV*, *VE7*, and *VE12*. Chemically induced mutations to *NtLs*, *NtBl1*, and *NtREV* similarly inhibited the development of secondary and “tertiary” lateral shoots, but not primary lateral shoots. The mutations to *NtLs* and *NtBl1* were incorporated into an elite variety by backcrossing. The agronomic characteristics of the backcross lines were examined in field trials conducted in commercial tobacco production regions. The lines were generally suitable for tobacco leaf production and may be useful as new tobacco varieties.

**Conclusion:**

The suppressed expression of *NtLs*, *NtBl1*, *NtREV*, *VE7*, or *VE12* inhibited the development of only the secondary and tertiary lateral shoots in tobacco. The mutant lines may benefit tobacco farmers by minimizing the work required to remove secondary and tertiary lateral shoots that emerge when farmers are harvesting leaves, which is a labor-intensive process.

**Supplementary Information:**

The online version contains supplementary material available at 10.1186/s12870-021-03008-3.

## Background

Lateral shoot development, which is a fundamental process in plants, is uniquely regulated in all species. In crop species, harvest quality and productivity are directly affected by how lateral shoot formation is controlled. Genes involved in axillary meristem development have been studied in various plant species. Mutations in *Lateral suppressor* genes in tomato (*Ls*) [1] and Arabidopsis (*LAS*) [2] inhibit axillary shoot formation during the vegetative phase. A mutation in a rice ortholog, *Monoculm 1* (*MOC1*), results in suppressed tillering and a decrease in the number of rachis branches and spikelets [3]. A mutation in the *Blind* gene strongly suppresses axillary meristem formation in tomato [4, 5]. Three *Regulator of Axillary Meristems* (*RAX*) genes, *RAX1*, *RAX2*, and *RAX3*, were identified in Arabidopsis as homologs of *Blind*. Triple recessive mutants exhibit almost completely inhibited axillary shoot formation [6, 7]. *Blind* and *RAX* genes encode R2R3 MYB transcription factors, which are critical for initiating axillary meristem development during the vegetative phase. Previous studies revealed that *Blind* also controls axillary meristem initiation in the reproductive phase [5, 6]. The decapitation of primary shoots does not stimulate the outgrowth of axillary shoots in the Arabidopsis *las* mutant or *rax1* mutant [2, 7]. The *LAX1* gene in rice and the *BA1* gene in maize encode bHLH domains and are involved in the branching of inflorescence and vegetative shoots [8, 9]. The *ROX* gene in Arabidopsis is an ortholog of *LAX1* and *BA1*. It is reportedly involved in lateral shoot formation in the early vegetative stage [10].

Mutations in some genes regulating axillary meristem formation affect the development of the main shoot apices. For example, a mutation in the *Revoluta* (*REV*) gene in Arabidopsis substantially decreases the outgrowth of rosette and cauline leaves and inhibits the stimulation of axillary shoots following the decapitation of primary shoots [11, 12]. Additionally, the *rev* mutation sometimes leads to the arrested development of the primary shoot apical meristem (SAM) at an early stage. The *Cup-Shaped Cotyledon* (*CUC*) genes *CUC1*, *CUC2*, and *CUC3* [13–15] and the *Lateral Organ Fusion* (*LOF*) gene [16] regulate organ separation and axillary meristem formation in Arabidopsis. *Hairy Meristem* (*HAM*) genes in petunia [17] and pepper [18] and their homolog in Arabidopsis, *Lost Meristem* (*LOM*) [19], are important for SAM and axillary meristem development. Consequently, petunia and pepper *ham* mutants and the Arabidopsis *lom1*-*lom2*-*lom3* triple mutant exhibit premature termination of the shoot apex and arrested axillary shoot development. A mutation in the *Far-Red Elongated Hypocotyl* (*FHY3*) gene in Arabidopsis leads to inhibited axillary bud outgrowth. Additionally, the *rev*-*fhy3* double mutation represses axillary bud formation considerably more than the single recessive mutations [20].

Because tobacco is cultivated for its leaves, when plants start to flower, the apical portions of stems (mainly inflorescences) are cut off (i.e., topping) to enhance leaf development and maturation prior to harvest. Although tobacco plants generally exhibit strong apical dominance, topping releases lateral buds from dormancy. The resulting lateral shoots, which are often called “suckers” in tobacco, undergo vigorous development. Tobacco farmers must remove these “primary” lateral shoots soon after they develop, but “secondary” lateral shoots grow immediately from the abaxial side of the primary lateral shoot base. Thus, lateral shoots emerge sequentially during the cultivation period (up to “tertiary” lateral shoots). These lateral shoots must be removed manually or suppressed by chemicals (i.e., “suckercides”) to enable plants to produce many high-quality leaves. However, this control of lateral shoots is a labor-intensive and costly process. Removing the secondary and tertiary lateral shoots is especially inconvenient because the timing of their emergence coincides with when farmers must harvest the leaves, which is another labor-intensive task. Therefore, tobacco varieties with fewer lateral shoots, especially secondary and tertiary ones, are highly desired. Unfortunately, breeding involving conventional and biotechnological approaches has not resulted in varieties with substantially fewer lateral shoots.

The tobacco genes involved in the regulation of axillary shoot development and their functions remain relatively uncharacterized. Nevertheless, it is possible that homologs of the above-mentioned genes in other plants are present and function similarly in tobacco. For example, a tobacco homolog (GenBank EU935981) of *Ls* has been cloned [21], but its function was not investigated. In this study, the expression of the homologs of these genes as well as a number of other genes preferentially expressed in the axillary meristems was knocked down in tobacco by RNA interference (RNAi). Additionally, the chemically induced mutations to selected genes inhibited lateral shoot formation. Mutant lines were characterized in greenhouse and field trials.

## Results

### BLAST search

Two groups of tobacco genes were examined to assess the effects of RNAi knock-down on lateral shoot formation. The first group included homologs of the genes involved in lateral shoot formation in other plants. A BLAST search of the GenBank database and an in-house tobacco cDNA database was conducted to identify tobacco homologs (Table 1). Because tobacco is an amphidiploid species that inherited its genome from *Nicotiana sylvestris* (S-genome) and *Nicotiana tomentosiformis* (T-genome) [22–24], both S-genes and T-genes were identified for each homolog. The *NtBl* and *NtCUC* genes were numbered respectively in descending order of homology with *Blind* and *Goblet* (GenBank HM210879), a tomato homolog of the *CUC* genes.

**Table 1:** Tobacco homologs of genes involved in the regulation or development of axillary meristems and shoots in other species

Query gene (Species, GenBank accession) for BLAST search	Tobacco homologue	Accession number in GenBank or Sol Genomics Network^a^	
		S-gene	T-gene
*Ls* (Tomato, AF098674)	*NtLs*	EU935581	AM848584
MYB domain of *Blind* (Tomato, AF426174.1)	*NtBl1*	Nitab4.5_0001442g0010.1	Nitab4.5_0004993g0020.1
	*NtBl2*	Nitab4.5_0001050g0010.1	Nitab4.5_0001163g0150.1
	*NtBl3*	Nitab4.5_0007679g0010.1	Nitab4.5_0000578g0120.1
*REV* (Tomato, BT013577 and Arabidopsis, AF233592)	*NtREV*	JQ686937	Nitab4.5_0004624g0050.1
NAC domains of *CUC* genes (Arabidopsis, NM_112380, NM_124774, and NM_106292)	*NtCUC1*	Nitab4.5_0007278g0010.1	Nitab4.5_0000568g0080.1
	*NtCUC2*	Nitab4.5_0008840g0010.1	Nitab4.5_0005914g0020.1
	*NtCUC3*	Nitab4.5_0004286g0020.1	Nitab4.5_0007189g0080.1
	*NtCUC4*	XM_016587094	XM_016602286.1
*FHY3* (Arabidopsis, NM_001125201)	*NtFHY3*	Nitab4.5_0002232g0050.1	XM_016593996.1
*LOM* (Arabidopsis, NM_130079.3)	*NtLOM1*	Nitab4.5_0011278g0010.1	XM_016625781.1
*LOF* (Arabidopsis, NM_001160897.2)	*NtLOF1*	XM_016601029.1	XM_016602279.1

The tobacco *Bl* and *CUC* homolog number was determined on the basis of the sequence similarity to a tomato *Bl* gene (AF426174.1) and *CUC* gene (HM210879.1), respectively

^a^Homologs originally identified in the in-house database were later submitted to the Sol Genomics Network database (https://solgenomics.net/) and GenBnak (https://www.ncbi.nlm.nih.gov/genbank/) by other research groups. Therefore, the accession numbers in the public database are provided

### RNA sequencing (RNA-seq) analysis

**Fig. 1:**
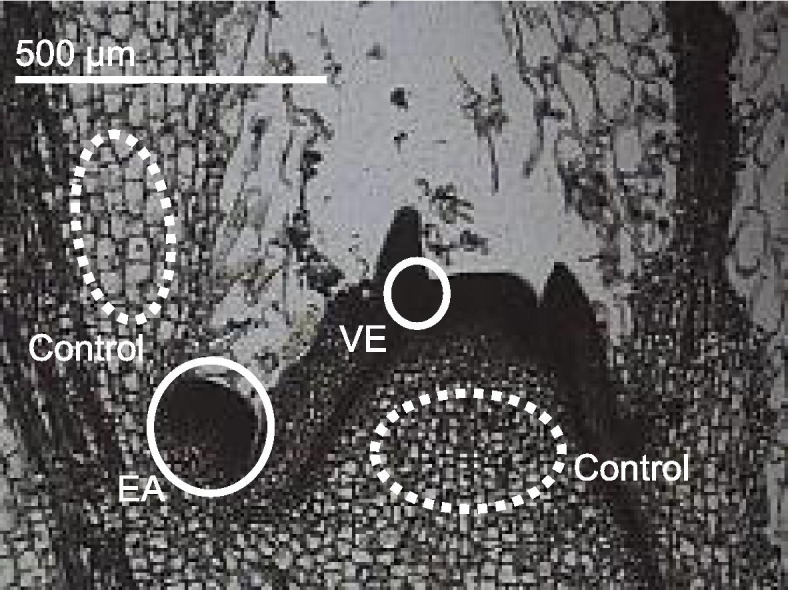
Tissues at early (EA) and very early (VE) axillary meristem developmental stages and control tissues sampled by laser micro-dissection

Genes that were not studied in other plants might also be involved in the regulation or development of axillary meristems and shoots. Thus, as the second group, genes that are highly expressed in axillary bud primordial stages were identified by RNA-seq. Tissues were sampled from axillary meristem zones in the early stage (EA) and the very early stage (VE) and from the control zones of tobacco plants by laser micro-dissection (Fig. 1). Total RNA was extracted from the tissues and analyzed using the 454 GS FLX next-generation sequencer (454 Life Sciences, Roche). Approximately 900,000 reads, with an average length of approximately 400 bp, were obtained for each tissue sample and were assembled into 38,569 unique genes. The second group of genes was selected according to the following criteria: (a) the read count of a gene in EA or VE was at least 10-times higher than that in the control, (b) the assembled gene was longer than 200 bp, and (c) the gene encodes a transcription factor or unknown protein according to the annotation. We selected 11 genes highly expressed in EA tissue (*EA1*–*EA11*) and 13 genes highly expressed in VE tissue (*VE1*–*VE13*) (Table 2).

**Table 2:** Expression of the genes in the early axillary meristem tissues and effects of RNA interference

Gene	RNAseq counts (Reads Per Kilobase of exon per Million mapped sequence reads)			Effect of RNAi measured relative to null segregants ± SD				
				RNA expression	Primary lateral shoots		Secondary lateral shoots	
	EA	VE	Control		Number	Weight	Number	Weight
*NtLs*	1.16	62.97	1.14	0.50 ± 0.08	1.00 ± 0.00	1.28 ± 0.15	0.13 ± 0.09^a^	0.1 ± 0.06^a^
*NtBl1*	12.80	108.85	8.43	0.31 ± 0.05	1.02 ± 0.06	1.21 ± 0.18	0.00 ± 0.00^a^	0.00 ± 0.00^a^
*NtBl2*	ND	ND	ND	0.19 ± 0.04	1.00 ± 0.00	1.13 ± 0.15	1.00 ± 0.10	1.46 ± 1.65
*NtBl3*	11.88	23.20	10.26	0.30 ± 0.09	1.00 ± 0.00	1.45 ± 0.48	0.88 ± 0.46	0.77 ± 0.12
*NtREV*	110.74	167.99	305.61	0.62 ± 0.10	1.00 ± 0.00	0.98 ± 0.09	0.06 ± 0.10^a^	0.03 ± 0.04^a^
*NtCUC1*	4.24	10.32	8.38	0.16 ± 0.07	1.00 ± 0.00	1.05 ± 0.12	0.91 ± 0.18	0.57 ± 0.10
*NtCUC2*	ND	ND	ND	0.53 ± 0.04	1.00 ± 0.00	1,14 ± 0.26	0.97 ± 0.12	1.06 ± 0.4
*NtCUC3*	ND	ND	ND	0.17 ± 0.10	1.00 ± 0.00	0.96 ± 0.17	1.08 ± 0.01	0.71 ± 0.26
*NtCUC4*	ND	ND	ND	0.69 ± 0.03	1.00 ± 0.00	0.91 ± 0.04	1.05 ± 0.09	0.75 ± 0.19
*NtFHY3*	23.49	36.90	46.59	0.31 ± 0.17	1.00 ± 0.00	0.94 ± 0.08	1.03 ± 0.11	1.12 ± 0.62
*NtLOM1*	36.69	49.15	65.87	0.38 ± 0.06	1.00 ± 0.00	1.09 ± 0.15	1.72 ± 1.97	2.56 ± 3.99
*NtLOF1*	30.27	60.57	25.56	0.38 ± 0.11	1.01 ± 0.02	1.67 ± 0.15	0.28 ± 0.30	0.64 ± 0.58
*EA1*	32.93	2.30	ND	0.09 ± 0.03	1.00 ± 0.00	1.14 ± 0.16	0.97 ± 0.15	1.21 ± 0.34
*EA2*	20.13	9.70	1.48	0.28 ± 0.16	1.00 ± 0.00	1.17 ± 0.35	1.34 ± 0.82	1.65 ± 1.59
*EA3*	22.56	1.77	ND	0.16 ± 0.03	1.00 ± 0.00	0.93 ± 0.09	0.68 ± 0.16	1.42 ± 1.53
*EA4*	43.22	2.50	1.19	0.35 ± 0.17	1.00 ± 0.00	1.16 ± 0.28	1.14 ± 0.41	1.09 ± 0.38
*EA5*	14.41	ND	ND	0.10 ± 0.04	1.00 ± 0.00	1.04 ± 0.07	4.05 ± 2.21	18.88 ± 28.34
*EA6*	22.78	ND	ND	0.80 ± 0.22	1.00 ± 0.00	1.01 ± 0.26	1.04 ± 0.27	1.30 ± 0.80
*EA7*	12.14	ND	ND	0.10 ± 0.01	1.00 ± 0.00	1.02 ± 0.17	0.83 ± 0.76	0.77 ± 0.70
*EA8*	43.57	ND	1.47	0.02 ± 0.02	1.00 ± 0.00	1.08 ± 0.36	1.14 ± 0.11	1.21 ± 0.2
*EA9*	14.03	ND	ND	0.17 ± 0.12	1.00 ± 0.00	0.95 ± 0.17	1.10 ± 0.77	1.16 ± 0.74
*EA10*	51.93	8.72	1.25	0.43 ± 0.08	1.00 ± 0.00	1.33 ± 0.16	1.59 ± 0.99	0.96 ± 0.21
*EA11*	19.63	1.70	1.62	0.56 ± 0.08	1.00 ± 0.00	0.98 ± 0.27	0.98 ± 0.09	1.52 ± 0.92
*VE1*	39.71	49.37	3.79	0.58 ± 0.06	1.00 ± 0.00	0.99 ± 0.30	1.10 ± 0.16	1.27 ± 0.51
*VE2*	26.70	32.21	0.69	0.51 ± 0.02	1.00 ± 0.00	1.04 ± 0.12	1.09 ± 0.17	0.95 ± 0.54
*VE3*	6.51	49.14	ND	1.51 ± 0.05	1.00 ± 0.00	1.16 ± 0.21	1.02 ± 0.45	0.97 ± 0.35
*VE4*	ND	10.94	ND	0.32 ± 0.04	1.00 ± 0.00	1.27 ± 0.31	0.80 ± 0.38	0.95 ± 0.64
*VE5*	20.88	79.27	3.12	0.41 ± 0.06	1.00 ± 0.00	1.00 ± 0.17	1.3 ± 0.74	1.41 ± 0.84
*VE6*	2.61	31.26	ND	0.35 ± 0.14	1.00 ± 0.00	0.73 ± 0.12	2.06 ± 0.56	4.57 ± 3.20
*VE7*	ND	73.71	4.97	0.08 ± 0.02	1.00 ± 0.00	1.49 ± 0.30	0.33 ± 0.09^a^	0.26 ± 0.11^a^
*VE8*	5.93	13.38	ND	0.14 ± 0.06	1.00 ± 0.00	1.01 ± 0.28	1.41 ± 0.43	0.87 ± 0.39
*VE9*	5.06	14.07	ND	0.62 ± 0.08	1.00 ± 0.00	0.98 ± 0.10	1.03 ± 0.16	0.90 ± 0.12
*VE10*	9.42	21.58	1.86	0.42 ± 0.04	1.00 ± 0.00	0.97 ± 0.25	1.16 ± 0.62	1.79 ± 1.29
*VE11*	ND	13.62	ND	0.70 ± 0.07	1.00 ± 0.00	1.07 ± 0.28	0.58 ± 0.56	0.42 ± 0.43
*VE12*	19.79	133.95	11.71	0.18 ± 0.06	1.00 ± 0.00	1.22 ± 0.12	0.29 ± 0.41	0.05 ± 0.08^a^
*VE13*	5.68	19.24	ND	0.14 ± 0.05	1.00 ± 0.00	1.03 ± 0.21	1.03 ± 0.12	1.86 ± 0.94

*ND* Not detectable

^a^Significantly different from null segregants at the 1% level as determined by the *t*-test, assuming that the means of the relative values were from normal distributions

The RNA read counts for the first group of genes (i.e., tobacco homologs) are listed in Table 2. A comparison with the control tissue indicated *NtLs* and *NtBl1* were highly and specifically expressed in EA or VE tissue. In all three samples, *NtREV* was highly expressed. Regarding the other homologs, there were no significant differences in the expression levels among the three tissues or the expression levels were too low to detect. Earlier investigations proved that *LAS* and *RAX* genes are highly expressed in the axil of leaf primordia [2, 6, 7]. Additionally, *REV* is reportedly expressed in the initial lateral shoot meristem as well as in the center of the SAM in an inverted-cup-shaped cell population [12]. Consequently, the expression patterns of at least some of the homologs (*NtLs*, *NtBl1*, and *NtREV*) were similar to those of their counterparts in other species.

Notably, the sequence analysis revealed that *VE7*, which encodes a bHLH domain, is a homolog of the *ROX* gene in Arabidopsis. The identification of the *ROX* was published after our BLAST search for homologs of the genes described above [10]. Additionally, *VE12*, which encodes a NAC domain and lacks a recognition site for miR164, was identified as a *CUC3* homolog. Previous studies confirmed that *ROX* [10] and its maize ortholog *BA1* [9] are expressed at the adaxial boundary of leaf primordia and that *CUC3* is expressed at the boundaries between leaf primordia and the shoot meristem [25]. Accordingly, *VE7* and *VE12* are similar to the homologs regarding their expression patterns. In addition to the early stage of axillary meristem, *Ls*, *Bl1*, and most of the *VE* and *EA* genes were expressed in other tissues and organs (Supplemental Table S1). More importantly, the RNA-seq analysis supplemented the search for homologs.

### RNAi knock-down

A total of 36 trigger sequences, with an average length of 430 bp, were designed for the RNA interference of the 12 homologs and 24 genes selected during the RNA-seq screening (Supplemental Table S2). Three *NtBl* and four *NtCUC* genes were obtained. Therefore, the less conserved regions (i.e., less than 70% identity) of the three *NtBl* genes and the four *NtCUC* genes were used for designing trigger sequences to distinguish each gene. For each target, the S-gene and T-gene were compared. A highly homologous region was chosen as the trigger to ensure that both genes were effectively knocked down by a single trigger sequence. The average sequence identity between the trigger regions of the S-genes and T-genes was 95.4%, which was higher than the homology sufficient for simultaneously knocking down two genes determined by Parrish et al. [26].

**Fig. 2:**
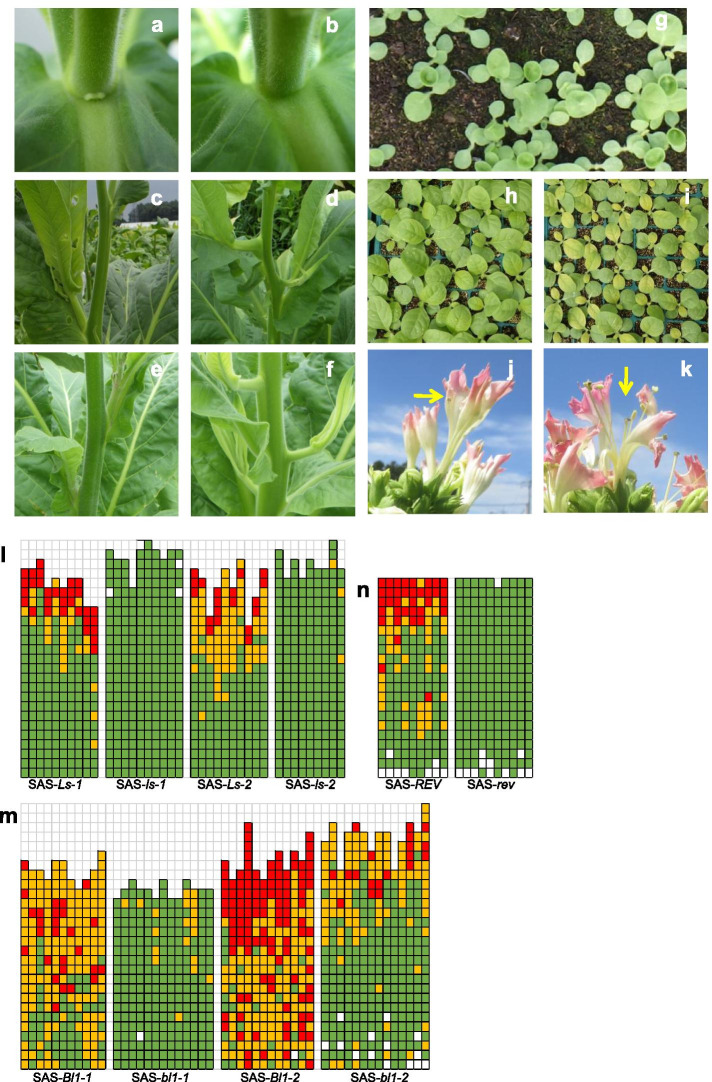
**a**, **b** Adaxial side of the primary lateral shoot base in a wild-type plant (**a**) and in an RNAi *NtLs* knock-down plant (**b**); **c**–**f** Primary lateral shoots of SAS-*Ls-1* (**c**), SAS-*Bl-1-1* (**e**), SAS*-ls-1* (**d**), and SAS-*bl1-1* (**f**) plants; **g**–**i** Seedlings of the SAS-*rev* mutant (**g**), ‘Coker319′ (**h**), and Coker319*-ls-1* (**i**); **j**, **k** Flowers of Coker319*-ls-1* plants; **l**–**n** Diagrammatic representation of primary, secondary, and tertiary lateral shoot formation in the leaf axils of SAS-*Ls* and *ls* lines (**l**), SAS-*Bl1* and *bl1* lines (**m**), and SAS-*REV* and *rev* lines (**n**). Each column in (**l**–**n**) represents a single plant, with each square representing an individual leaf axil. From bottom to top, the squares represent the leaf positions of plants after topping. Red indicates the presence of primary to tertiary (or more) lateral shoots; yellow indicates the presence of primary to secondary lateral shoots; green indicates the presence of only the primary lateral shoot; and white indicates a lack of lateral shoots

The RNAi genes were introduced into *Nicotiana tabacum* L. cv. Petit Havana SR-1. The T_1_ progeny of three single-locus transformants for each construct were grown in a greenhouse and were examined regarding target gene expression. The target genes were thoroughly suppressed in the analyzed plants (Table 2), with the exception of the *VE3* transformants, in which the target gene was more highly expressed than in the null segregants; the reasons for the increased expression remain unknown. The data revealed a lack of difference in primary lateral shoot production (i.e., number and weight) between the transgenic plants and the null segregants (Table 2). Therefore, the knock-down of target gene expression by RNAi did not suppress primary lateral shoot formation. However, the production of secondary lateral shoots was suppressed in terms of number and/or weight in the transformants in which *NtLs*, *NtBl1*, *NtREV*, *VE7*, and *VE12* were targeted. For example, a secondary lateral bud was visible at the base of the abaxial side of a primary lateral shoot in wild-type plants (Fig. 2a), but not in transgenic plants in which *NtLs* expression was knocked down (Fig. 2b). After the secondary lateral shoots were removed, no additional lateral shoots were formed on plants grown in the greenhouse. An earlier investigation indicated that a sequence match of at least 22 consecutive nucleotides may result in off-target silencing by a trigger sequence [27]. Therefore, we performed a BLAST search to screen for 22 consecutive nucleotides identical to the trigger sequences designed for the five effective genes (i.e., *NtLs*, *NtBl1*, *NtREV*, *VE7*, and *VE12*). With the exception of *VE12*, there were no tobacco genes with 22 consecutive nucleotides identical to the trigger sequences. The *VE12* gene is considered to be a NAC gene, which belongs to one of the largest transcription factor families in tobacco [28]. Therefore, we cannot exclude the possibility of off-target silencing in the *VE12*_RNAi tobacco plants. Regarding *NtBl1*, we confirmed that the expressions of *NtBl2* and *NtBl3* were not down-regulated in the *NtBl1*_RNAi tobacco plants. Therefore, the RNAi results were validated further.

### Chemically induced mutants

**Fig. 3:**
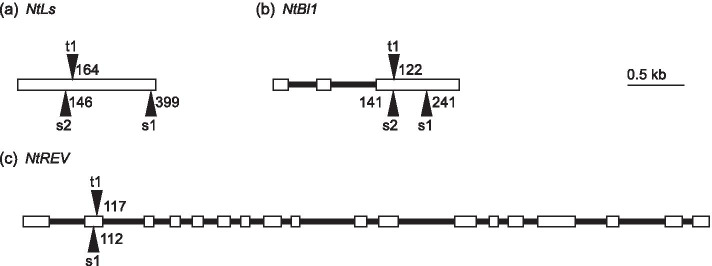
Locations of chemically induced loss-of-function mutations. Numbers indicate the positions of the nonsense codons from the start of the open reading frame in **a*** NtLs*, **b*** NtBl1* and **c*** NtREV*. Exons and introns are represented by open boxes and black lines, respectively

The ethyl methanesulfonate (EMS) mutant library for *Nicotiana tabacum* L. cv. Tsukuba 1 was screened for nonsense mutations in *NtLs*, *NtBl1*, *NtREV*, *VE7*, and *VE12*. The following mutations were identified and mapped (Fig. 3): two each in *NtLs-S* and *NtBl1-S* and one each in *NtLs-T*, *NtBl1-T*, *NtRev-S*, and *NtRev-T*. Moreover, mutations in the S-genome and T-genome were combined by crossing the mutants, producing double-mutant lines designated as SAS lines (Table 3). Because nonsense mutations were not identified in *VE7* and *VE12*, these genes were not studied further.

**Table 3:** Mutant lines and wild-type segregants characterized in field trials

Line code	Target gene	Genotype	Generation
SAS-*ls-1*	*NtLs*	*s1s1/t1t1*	(M2 × M2) F3
SAS-*Ls-1*	*NtLs*	*SS/TT*	(M2 × M2) F3, wild type segregant
Coker319-*ls-1*	*NtLs*	*s1s1/t1t1*	((Coker319 x (M2 x M2) F1) x Coker319) BC6F3
SAS-*ls-2*	*NtLs*	*s2s2/t1t1*	(M2 × M2) F3
SAS-*Ls-2*	*NtLs*	*SS/TT*	(M2 × M2) F3, wild type segregant
SAS-*bl1-1*	*NtBl1*	*s2s2/t1t1*	(M2 × M2) F3
SAS-*Bl1-1*	*NtBl1*	*SS/TT*	(M2 × M2) F3, wild type segregant
Coker319-*bl1-1*	*NtBl1*	*s2s2/t1t1*	((Coker319 × (M2 × M2) F1) × Coker319) BC6F3
SAS-*bl1-2*	*NtBl1*	*s1s1t1t1*	(M2 × M2) F3
SAS-*Bl1-2*	*NtBl1*	*SS/TT*	(M2 × M2) F3, wild type segregant
SAS-*rev*	*NtREV*	*s1s1/t1t1*	(M2 × M2) F3
SAS-*REV*	*NtREV*	*SS/TT*	(M2 × M2) F3, wild type segregant

Mutations in some genes involved in axillary meristem formation reportedly affect the development of the main shoot apices [11–19]. The seedlings of the SAS-*rev* mutant lines produced cup-shaped or asymmetric cotyledons (Fig. 2g). Additionally, the leaves of 4- to 5-week-old Coker319-*ls-1* plants*,* backcross line for field trials at commercial production sites, grown in nursery boxes occasionally turned yellow (Fig. 2i). The same phenotype was observed in 4- to 5-week-old Coker319-*ls-1* plants incubated at 15 °C in a growth chamber, although the plants appeared normal at 28 °C. These findings suggest that Coker319-*ls-1* leaf coloration may be sensitive to cold stress. However, the yellowed plants recovered quickly, with no delayed growth. Normally growing SAS lines were transferred to an experimental field for an examination of their lateral shoot development. Again, the primary lateral shoots were not significantly suppressed in the SAS-*ls*, SAS-*bl1*, and SAS-*rev* plants (Table 4), whereas the number and weight of secondary lateral shoots decreased. Furthermore, tertiary lateral shoot formation was nearly completely suppressed (Fig. 2l, m, and n). The phenotypes of the RNAi lines in the greenhouse were consistent with the phenotypes of the EMS mutants in the field. Interestingly, the position of the primary lateral shoots shifted upward in the SAS-*ls* and SAS-*bl1* mutant lines (Fig. 2d and f) in the field and greenhouse (data not shown). A similar shift was not observed in the RNAi lines grown in the greenhouse (Fig. 2b). The positions of the secondary and tertiary lateral shoots in the mutant lines and the RNAi lines were unchanged. Only the position of the primary lateral shoots in the mutant lines of *NtLs* and *NtBl1* was affected, but the reasons for this shift remain unclear.

**Table 4:** Lateral shoot production in field trials at an experimental site

Trial No	Line code	Primary lateral shoots		Secondary lateral shoots		Tertiary lateral shoots	
		Number ± SD	Weight (g) ± SD	Number ± SD	Weight (g) ± SD	Number ± SD	Weight (g) ± SD
1	SAS-*Ls-1*	20.60 ± 1.65	36.79 ± 15.34	5.60 ± 1.78	9.99 ± 5.09	3.10 ± 0.99	4.27 ± 2.46
	SAS-*ls-1*	23.10 ± 1.37	18.42 ± 7.09	0.00 ± 0.00^a^	0.00 ± 0.00^a^	0.00 ± 0.00^a^	0.00 ± 0.00^a^
	SAS-*Ls-2*	20.20 ± 2.30	37.44 ± 18.29	8.40 ± 2.55	22.33 ± 9.33	1.90 ± 1.10	3.71 ± 3.21
	SAS-*ls-2*	22.56 ± 1.33	32.03 ± 11.72	0.33 ± 0.71^a^	0.50 ± 1.10^a^	0.00 ± 0.00^a^	0.00 ± 0.00^a^
2	SAS-*Bl1-1*	21.10 ± 1.22	57.38 ± NC	16.00 ± 3.61	64.98 ± NC	2.30 ± 1.75	17.67 ± NC
	SAS-*bl1-1*	18.80 ± 0.80	42.87 ± NC	1.46 ± 2.18^a^	8.67 ± NC	0.00 ± 0.00^a^	0.00 ± NC
	SAS-*Bl1-2*	22.00 ± 1.71	155.01 ± NC	19.83 ± 2.86	56.13 ± NC	12.17 ± 5.86	57.02 ± NC
	SAS-*bl1-2*	23.60 ± 2.28	95.94 ± NC	8.36 ± 3.82^a^	49.57 ± NC	1.43 ± 1.70^a^	5.38 ± NC
3	SAS-*REV*	19.89 ± 0.60	85.11 ± 39.78	8.78 ± 2.91	35.63 ± 12.48	5.33 ± 2.96	17.10 ± 14.11
	SAS-*rev*	19.80 ± 0.63	78.42 ± 29.98	0.00 ± 0.00^a^	0.00 ± 0.00^a^	0.00 ± 0.00^a^	0.00 ± 0.00^a^

*NC* Not calculable

^a^Significantly different from the wild-type line(s) in the same trial at the 1% level as determined by the *t*-test

### Field trials in commercial production areas

Field trials in regions used for the commercial production of tobacco were conducted to examine the agronomic characteristics of the mutant lines. The *ls-1* and *bl1-1* mutations were introduced into a flue-cured tobacco variety ‘Coker319′ widely cultivated in Japan (*Nicotiana tabacum* L. cv. Coker319) via backcross breeding (Table 3). Because of space limitations, the *rev* mutation was not tested.

The results of the analyses of Coker319-*ls-1* at four locations and Coker319-*bl1*-*1* at two locations are presented in Table 5. A plot comprising 10 plants was replicated twice for each line at all locations. Overall, the mutant lines did not differ much from the original ‘Coker319′ plants in terms of leaf yield or other parameters, although small, but statistically significant, differences were detected. Specifically, compared with ‘Coker319′ plants, the Coker319-*ls-1* plants at one or more locations were taller, had larger (length and width) and darker leaves, and flowered later. In contrast, the Coker319-*bl1*-*1* plants at one or two locations were shorter and produced more leaves, which were smaller and less intensely colored (at 1/2 from the top), than the ‘Coker319′ plants. Therefore, the mutant lines were appropriate for the subsequent trials. Additionally, the development of lateral shoots, especially the secondary and tertiary lateral shoots, was inhibited. A preliminary survey revealed that the labor necessary for removing lateral shoots was 52% lower for Coker319-*ls-1* plants than for ‘Coker319′ plants (Supplemental Table S3).

**Table 5:** Agronomic characteristics in field trials at commercial production sites

Location	Line	Days to flower	Plant height (cm)	Number of leaves	Leaf at 1/4 from the top			Leaf at 1/2 from the top			Leaf at 3/4 from the top			Leaf yield^d^ (kg/10 a)
					Length (cm)	Width (cm)	Color scale^c^	Length (cm)	Width (cm)	Color scale^c^	Length (cm)	Width (cm)	Color scale^c^	
1	Coker319	69.3	128.0	19.9	43.5	12.0	8.2	56.0	20.5	6.4	56.1	27.5	5.8	281.6
	Coker319-*ls*-*1*	69.9^a^	134.1^b^	20.2	45.2	14.0^b^	8.2	57.1	23.3^b^	6.9^b^	56.6	30.3^b^	6.0	305.3
2	Coker319	72.8	110.5	22.1	48.7	13.1	7.5	66.6	20.9	7.4	59.8	28.6	7.0	191.2
	Coker319-*ls*-*1*	73.5^a^	115.0^a^	22.5	49.9	13.8	7.2	65.8	23.4^b^	7.1	56.2^b^	28.8	6.9	253.1
3	Coker319	69.1	132.5	19.4	44.8	10.9	8.0	55.5	19.0	7.1	58.3	27.1	6.3	268.5
	Coker319-*ls*-*1*	69.9^a^	133.0	19.6	48.5^b^	13.3^b^	8.0	59.2^b^	23.3^b^	7.7^b^	59.5	30.1^b^	6.8^b^	271.0
4	Coker319	59.6	134.3	17.1	60.0	18.3	8.9	69.7	25.0	8.8	65.4	29.2	8.0	236.9
	Coker319-*ls*-*1*	61.0^b^	131.6	18.3^a^	63.4^a^	22.9^b^	9.0	72.2	29.4^b^	8.9	62.4	29.3	8.2	267.5
5	Coker319	63.3	129.8	19.1	50.8	14.0	8.4	62.5	24.9	7.8	58.4	27.8	6.5	218.0
	Coker319-*bl1*-*1*	63.9	119.5^b^	20.7^b^	50.1	13.4	8.7	61.8	23.1^a^	7.9	52.2	27.2	6.6	218.0
6	Coker319	65.7	114.3	22.4	37.1	11.5	7.5	52.8	18.6	7.7	56.3	27.3	6.4	293.0
	Coker319-*bl1*-*1*	66.2	116.3	24.7^b^	35.1	11.1	7.4	54.0	17.3	7.5	57.3	28.5	6.1^a^	288.0

^a^, ^b^Significantly different from wild-type ‘Coker319′ at the same location at the 5% and 1% levels, respectively, as determined by the *t*-test

^c^Higher numbers indicate darker color

^d^Yield of cured leaves

### Flower development and fertility

In the greenhouse and field, the petals of most of the flowers of SAS-*ls-1*, SAS-*ls-2*, and Coker319*-ls-1* plants were split (Fig. 2j). In severe cases, pistils and stamens were completely exposed (Fig. 2k). As compared to naturally pollinated WT flowers, the seed yield from the abnormal flowers of Coker319-*ls-1* was decreased by 35% upon natural pollination and 55% upon hand pollination (Supplemental Fig. S1).

The Coker319-*bl1-1* plants produced 73% fewer flowers than the wild-type plants in the field trials (Supplemental Fig. S2). The seed yield per flower of Coker319-*bl1-1* was 28% less than Coker319 (Supplemental Fig. S1).

## Discussion

Genetic inhibition of lateral shoot formation was attempted in tobacco. Two groups of tobacco genes were selected to examine the effects of RNAi on lateral shoot formation. One set of candidate genes comprised homologs of the genes reportedly involved in the formation of lateral shoots in other plants. Such genes have been studied most extensively in Arabidopsis. The following eight genes or gene families were identified: *LAS* [2], *RAX* family [6, 7], *ROX* [10], *REV* [11, 12], *CUC* family [13–15], *LOF* [16], *LOM* [19], and *FHY3* [20]. A BLAST search identified tobacco homologs for these genes, with the exception of *ROX*, which was reported after the BLAST search was performed. The *VE7* gene, which is homologous to *ROX*, was included in another gene set consisting of genes revealed to be highly expressed in the primordial stages of tobacco axillary buds by an RNA-seq analysis. A homolog of *CUC3*, *VE12*, was also in the second gene set. Therefore, tobacco and Arabidopsis apparently share similar sets of genes.

Transgenic lines in which *NtLs*, *NtBl1*, *NtREV*, *VE7*, or *VE12* expression was knocked down by RNAi as well as lines with chemically induced mutations to *NtLs*, *NtBl1*, or *NtREV* exhibited inhibited secondary lateral shoot formation. The *NtLs*, *NtBl1*, *NtREV*, *VE7*, and *VE12* genes are respectively homologs of *LAS*, *RAX1*, *REV*, *ROX*, and *CUC3*. Their reported expression patterns are consistent with the RNA-seq data presented herein. Additionally, mutations in *LAS*, *Ls* (in tomato), and *NtLs* affect flower morphology. The stamens of *las* mutant plants are abnormally short [2]. Other studies proved that petal development is suppressed in *ls* mutants and that the petals are mostly smaller than the wild-type petals [1, 29]. Moreover, decreased pollen production and seed production were commonly observed. Mutations in *Blind* [5], which is a tomato ortholog of *RAX1*, and *NtBl1* result in a decrease in the number of flowers. Therefore, *NtLs*, *NtBl1*, *NtREV*, *VE7*, and *VE12* might be considered respectively as the orthologs of *LAS*, *RAX1*, *REV*, *ROX*, and *CUC3*.

Some of the observed characteristics of the knock-down and chemically induced mutant tobacco plants were not reported for other plants. Tobacco primary lateral shoot formation was not suppressed, in contrast to the inhibited development of secondary and tertiary lateral shoots. Furthermore, the positions of the primary lateral shoots shifted upward in the tobacco mutants of *NtLs* and *NtBl1*. The mechanisms underlying the differences between plant species remain unclear; however, these differences are not surprising because the diversity in the regulation of lateral branching is a key factor associated with the considerable variety of plant shapes.

The other analyzed tobacco homologs were ineffective in the knock-down study. The expression of these genes was either undetectable or was not specific to the axillary bud primordial stages. They were probably nonfunctional homologs of genes in other plant species.

The candidate genes in the second set were highly expressed in the axillary bud primordial stages. Twenty-four genes were selected on the basis of the RNA-seq analysis. They were highly and specifically expressed in the EA or VE tissues. However, with the exception of *VE7* and *VE12*, knocking down the expression of these genes did not significantly inhibit lateral shoot formation. Accordingly, these genes might not have crucial regulatory roles related to lateral shoot development. Alternatively, their functions might be redundant in tobacco.

Two mutant tobacco lines with the ‘Coker319′ genetic background were examined in field trials at commercial tobacco production sites. These lines were satisfactory for tobacco leaf production and the trials should be repeated. Regarding the consequences of the inhibition of secondary and tertiary lateral shoot formation, the labor required for removing lateral shoots was assessed in a preliminary survey. Although cold sensitivity at the seedling stage and some floral abnormalities that decreased seed yield were observed in the *NtLs-*mutated plants, these characteristics are unlikely to affect tobacco leaf production practices.

The ‘Coker319′ derivatives examined in this study are potentially useful new tobacco varieties. They may decrease the labor and time required by tobacco growers to control secondary and tertiary lateral shoot formation. Additionally, breeding other varieties with mutations in *NtLs* and/or *NtBl1* and further characterizing the *NtLs*, *NtBl1*, *NtREV*, *VE7*, and *VE12* genes are among the important objectives that should be pursued in future studies.

## Conclusions

In this study, we attempted to develop tobacco lines with inhibited lateral shoot formation. There is a considerable demand for such lines among tobacco farmers. The RNA interference of five genes and the chemically induced mutations to three genes significantly suppressed lateral shoot development in tobacco under greenhouse and field conditions. Two mutant lines were evaluated regarding their agronomic performance in field trials conducted at commercial production sites. Although primary lateral shoot formation was not inhibited in the examined lines, the decreased production of secondary and tertiary lateral shoots may have implications for improved commercial tobacco production.

## Methods

### Plant materials

*Nicotiana tabacum* L. cv. Petit Havana SR-1 was provided by Fukui Prefectural University in 1995 and used for RNA sequencing and RNA interference. *Nicotiana tabacum* L. cv. Tsukuba 1 was developed at Japan Tobacco Inc. in 1980 and used for cloning of genes and development for chemically induced mutants. *Nicotiana tabacum* L. cv. Coker319 was provided by Coker’s Pedigreed Seed Co. in 1963 and used for breeding.

### BLAST analysis

The genes used as queries for BLAST searches of the NCBI BLAST database (http://blast.ncbi.nlm.nih.gov/Blast.cgi) and a database comprising tobacco cDNA sequences accumulated by a number of in-house studies are listed in Table 1.

### RNA sequencing

Apical tissues of shoots were collected from ‘Petit Havana SR-1′ tobacco seedlings at 29–37 days after germination, after which they were fixed in an ice-cold 3:1 solution of ethanol and acetic acid. The protocols used to prepare paraffin-embedded sections and laser-microdissection were previously described by Takahashi et al. [30]. Total RNA extracted using the PicoPure™ RNA isolation Kit (Thermo Fisher Scientific Inc.) was examined using the Agilent 2100 Bioanalyzer and the RNA 6000 Pico kit (Agilent Technologies Inc.). Next, cDNA was synthesized using an oligo-dT primer with the T7 promoter sequence, after which antisense RNA was transcribed using T7 RNA polymerase. The antisense RNA was served as the template for synthesizing cDNA using random primers. After a fragmentation step, the cDNA (400–1,000 bp) containing the 3′ end was collected and the 5′ end of the fragments were sequenced using the 454 GS FLX system. Files containing these sequences have been deposited at DDBJ (DRA011900). The obtained reads were assembled using Newbler (version 2.6).

### Gene cloning

After extracting coding sequence information from the GenBank and in-house cDNA databases, 5′- or 3′-RACE was performed using the SMARTer RACE cDNA Amplification Kit (Clontech) to obtain full-length cDNA clones of the coding sequences. Total RNA was extracted from dormant axillary buds, shoot apices, flower buds, or roots (Magtration®; Precision System Science Co., Ltd.). Amplified fragments were sequenced using the BigDye® (version 3.1) Cycle Sequencing Kit (Thermo Fisher Scientific Inc.) and the 3730xl DNA Analyzer (Thermo Fisher Scientific Inc.). Standard procedures were used for DNA manipulations [31]. Information regarding the obtained sequences is provided in Supplemental Table S4.

### RNA interference

The primers listed in Supplemental Table S2 were used to amplify trigger sequences from the cloned cDNA or RNA isolated from dormant axillary buds, shoot apices, flower buds, or roots collected from ‘Petit Havana SR-1′ tobacco plants. The amplified fragments were cloned into the pENTR™/D-TOPO vector (Takara Bio) and then introduced into a modified pHellsgate12 binary vector [32] containing an expression cassette comprising a gene encoding the green fluorescent protein under the control of the CaMV 35S promoter. The binary vectors were inserted into *Agrobacterium tumefaciens* strain LBA4404 cells, which were then used to transform ‘Petit Havana SR-1′ tobacco plants. The transgene locus number was determined on the basis of the segregation ratios for the green fluorescence in the T_1_ generation. Target gene expression levels were analyzed by quantitative PCR. Briefly, RNA extracted from the leaves, roots, or above-ground tissues excluding leaves was used as the template for synthesizing cDNA using the PrimeScript RT reagent kit with gDNA Eraser (Takara Bio). The quantitative PCR analysis was performed using the TaqMan Fast Advanced Master Mix (Thermo Fisher Scientific Inc.) and the StepOnePlus system (Thermo Fisher Scientific Inc.). Details regarding the primers and probes are listed in Supplemental Table S5. The gene encoding elongation factor-1α (AF120093) was used as the internal reference control. The relative expression of the target genes in the RNAi lines was compared with the expression in null segregant controls lacking the transgene.

### Chemically induced mutants

A library for *Nicotiana tabacum* L. cv. Tsukuba 1 mutated with EMS by Tajima et al. [33] was screened as described by Takakura et al. [34] using the primers listed in Supplemental Table S6.

### Plant cultivation

Tobacco plants were grown in pots (12 cm) containing soil in a greenhouse at 25 °C under natural day-length conditions or in a growth chamber with a 12-h light (25 °C):12-h dark (18 °C) cycle. They were also grown in an experimental field at the Leaf Tobacco Research Center of Japan Tobacco Inc. or in fields used for commercial tobacco production in southwestern Japan. The fields were managed according to the respective standard practices. When flower buds emerged, the plants were topped off, after which the lateral shoots were removed and measured weekly for 5 weeks in the growth chamber or for 8 weeks in the field. Leaf color was scored using the Leaf Color Chart 2019A for tobacco (Fujihira Industry; Supplemental Table S7).

## Supplementary Information

**Additional file 1: Supplemental Table S1.** Expression of genes in several tissues and organs. **Supplemental Table S2.** Primers for amplifying trigger DNA. **Supplemental Table S3.** Labor hours for manually removing lateral shoots in field trials at commercial production sites. **Supplemental Table S4.** Accession numbers of genes screened by RNA-seq. **Supplemental Table S5.** Primers and probes for quantitative PCR. **Supplemental Table S6.** Primers for screening mutations. **Supplemental Table S7.** CIELAB color scores (International Commission on Illumination; http://cie.co.at/) determined using Leaf Color Chart 2019A for tobacco.

**Additional file 2: Supplemental Fig. S1.** Seed yield of mutant lines. Statistical significance was determined using the *t*-test (**p < 0.01). NS: not significant.

**Additional file 3: Supplemental Fig. S2.** Number of flowers in Coker319*-bl1-1 *plants. Statistical significance was determined using the *t*-test (**p < 0.01).

## Data Availability

All data generated or analyzed during this study are included in the manuscript and its supplementary files. All images depicted in the figure are our own. The sequence information of tomato, Arabidopsis and tobacco genes is available from Solanaceae Genomics Network (https://solgenomics.net/), GenBank (https://www.ncbi.nlm.nih.gov/genbank/) and DNA DataBank of Japan (https://www.ddbj.nig.ac.jp/index-e.html). The raw reads of next-generation sequencing-based obtained in this study is available from the DDBJ/EMBL/NCBI under the accession number DRA011900 (https://ddbj.nig.ac.jp/DRASearch/submission?acc=DRA011900). The datasets generated or analyzed during the current study are available from the corresponding author for non-commercial purposes.

## Abbreviations

*Ls*, *LAS*: *Lateral suppressor*
*MOC1*: *Monoculm* *1*
*RAX*: *Regulator of axillary meristems*
MYB: Myeloblastosis
*LAX*: *Lax panicle*
*BA1*: *Barren stalk 1*
bHLH: Basic helix-loop-helix
*ROX*: *Regulator of axillary meristem formation*
*REV*: *Revoluta*
*CUC*: *Cup-shaped cotyledon*
*LOF*: *Lateral organ fusion*
*HAM*: *Hairy meristem*
*LOM*: *Lost meristem*
*FHY3*: *Far-red elongated hypocotyl3*
RNAi: RNA interference
NCBI: National Center for Biotechnology Information
BLAST: Basic local alignment search tool
*Bl*: *B* *lind*
RNA-seq: RNA sequencing
SAM: Shoot apical meristem
NAC: Petunia NAM and Arabidopsis ATAF1, ATAF2, and CUC2
EMS: Ethyl methanesulfonate
RACE: Rapid amplification of cDNA ends
CaMV: Cauliflower mosaic virus
SD: Standard deviation
