# Supplementary material for: Inhibition of lateral shoot formation by RNA interference and chemically induced mutations to genes expressed in the axillary meristem of Nicotiana tabacum L
Source: BMC Plant Biol. 2021 May 27;21:236. doi: 10.1186/s12870-021-03008-3 (PMC8157709; doi:10.1186/s12870-021-03008-3)
Supplement: Supplementary file 1 — Additional file 1: Supplemental Table S1. Expression of genes in several tissues and organs. Supplemental Table S2. Primers for amplifying trigger DNA. Supplemental Table S3. Labor hours for manually removing lateral shoots in field trials at commercial production sites. Supplemental Table S4. Accession numbers of genes screened by RNA-seq. Supplemental Table S5. Primers and probes for quantitative PCR. Supplemental Table S6. Primers for screening mutations. Supplemental Table S7. CIELAB color scores (International Commission on Illumination; http://cie.co.at/) determined using Leaf Color Chart 2019A for tobacco. [file 12870_2021_3008_MOESM1_ESM.docx]

Supplemental Table S1. Expression of genes in several tissues and organs

| Gene | Relative expression level (EF-1-alpha expression level=1) | | | | | | | | | | | | | | | | | |
| --- | --- | --- | --- | --- | --- | --- | --- | --- | --- | --- | --- | --- | --- | --- | --- | --- | --- | --- |
|  | Seedling | | Shoot Apex | | Stem | | Lamina | | Midrib | | Root | | Flower Bud | | Flower | | Axillary bud | |
| *NtREV1* | | 0.436 | | 0.516 | | 0.937 | | 0.484 | | 0.653 | | 0.603 | | 0.809 | | 0.879 | | 0.841 |
| *NtLS* | | 0.023 | | 0.020 | | 0.015 | | ND | | 0.011 | | 0.036 | | 0.047 | | 0.031 | | 0.056 |
| *NtBl1* | | 0.018 | | 0.010 | | 0.004 | | ND | | 0.002 | | 0.044 | | ND | | 0.010 | | 0.006 |
| *VE1* | | 0.145 | | 0.043 | | 0.131 | | 0.245 | | 0.052 | | 0.076 | | 0.069 | | 0.048 | | 0.181 |
| *VE2* | | 0.891 | | 0.772 | | 0.441 | | ND | | 0.384 | | 0.137 | | 1.585 | | 1.187 | | 0.712 |
| *VE3* | | ND | | ND | | ND | | ND | | ND | | ND | | ND | | ND | | ND |
| *VE4* | | 0.054 | | ND | | ND | | ND | | ND | | 0.337 | | 0.001 | | 0.006 | | ND |
| *VE5* | | 0.002 | | 0.008 | | 0.006 | | ND | | 0.007 | | 0.001 | | 0.013 | | 0.005 | | 0.014 |
| *VE6* | | ND | | 0.003 | | 0.024 | | ND | | 0.020 | | 0.008 | | 0.017 | | 0.012 | | ND |
| *VE7* | | 0.003 | | 0.003 | | ND | | ND | | ND | | ND | | ND | | 0.060 | | ND |
| *VE8* | | ND | | ND | | ND | | ND | | ND | | ND | | 0.062 | | 0.003 | | ND |
| *VE9* | | 0.491 | | 0.010 | | 0.008 | | 2.468 | | 0.153 | | 0.059 | | 0.031 | | 0.034 | | 0.013 |
| *VE10* | | 0.349 | | 0.008 | | 0.035 | | ND | | 0.002 | | 1.287 | | 0.012 | | 0.007 | | 0.013 |
| *VE11* | | 0.023 | | 0.055 | | 0.008 | | 0.011 | | 0.008 | | ND | | 0.101 | | 0.013 | | 0.033 |
| *VE12* | | 0.076 | | 0.165 | | 0.006 | | ND | | 0.017 | | ND | | 0.525 | | 0.115 | | 0.124 |
| *VE13* | | 0.001 | | 0.002 | | 0.072 | | 0.005 | | 0.007 | | 0.026 | | 0.005 | | 0.003 | | 0.003 |
| *EA1* | | ND | | 0.007 | | 0.018 | | ND | | 0.021 | | ND | | ND | | ND | | 0.194 |
| *EA2* | | 0.015 | | 0.023 | | 0.012 | | 0.006 | | 0.010 | | 0.029 | | 0.038 | | 0.007 | | 0.016 |
| *EA3* | | ND | | ND | | ND | | 0.001 | | ND | | ND | | 0.027 | | 0.003 | | ND |
| *EA4* | | 0.009 | | ND | | ND | | 0.030 | | ND | | ND | | ND | | 0.002 | | 0.008 |
| *EA5* | | 0.016 | | ND | | 0.040 | | 0.098 | | 0.099 | | 0.033 | | 0.007 | | 0.014 | | 0.013 |
| *EA6* | | 0.007 | | 0.013 | | 0.005 | | 0.055 | | 0.004 | | 0.004 | | 0.023 | | 0.034 | | 0.033 |
| *EA7* | | 0.008 | | 0.015 | | 0.013 | | 0.002 | | 0.003 | | 0.006 | | 0.064 | | 0.089 | | 0.043 |
| *EA8* | | 0.083 | | 0.066 | | 0.103 | | 0.062 | | 0.214 | | 0.015 | | 0.002 | | ND | | 0.017 |
| *EA9* | | ND | | ND | | ND | | 0.001 | | ND | | ND | | 0.007 | | 0.003 | | ND |
| *EA10* | | 0.001 | | ND | | ND | | ND | | ND | | 0.002 | | 0.005 | | 0.001 | | ND |
| *EA11* | | 0.018 | | 0.005 | | 0.042 | | 0.009 | | 0.024 | | 0.097 | | 0.012 | | 0.004 | | 0.005 |

ND: Not determined (relative expression level is less than 0.001)

Seedling: above-ground part at 10 days after seeding; Shoot Apex: top of young plants at 4 weeks after seeding; Stem: stem of young plants at 4 weeks after seeding; Lamina and Midrib: third and fourth leaves at 4 weeks after seeding; Root: root at 5 weeks after seeding; Axillary Bud: axillary bud of the upper leaf just before topping

EF-1-alpha: Accession No. AF120093

Supplemental Table S2. Primers for amplifying trigger DNA

| Gene | Forward primer (5′-3′) | Reverse primer (5′-3′) |
| --- | --- | --- |
| *NtLs* | CACCGAAGAAACTGATGATCAACGG | TCGCTTGATTAGCAGTCAGC |
| *NtBl1* | CACCTCAAGAAAAAGCTTATGGG | GCAGCAGCTAACAAGTTGTA |
| *NtBl2* | CACCTGGGCAGTTGTTTACAGAGTC | TTCAATTCCATCACAAGTACACA |
| *NtBl3* | CACCGGGATTTGTCTCTTCATCTCAGAAAATCAGGCC | GTGGGTCCCACTACTACAACTTCTTCAGA |
| *NtREV* | CACCGCCTATGTAGCTTCGTCAATG | CACTGTAGCCAGAGACCACA |
| *NtCUC1* | AGGGAAAAAGATAGCAATCTCAAGTC | ACCTCTCATATGATTGTTCTGTTTTG |
| *NtCUC2* | TTTCAGGGCTTATGCAATCAAACTC | TCAGCAACCCCAGAGGCAGTCAAGG |
| *NtCUC3* | ATACTAGCAAGATCAAGCCTTCTG | TCAGCAACCCCAGAGGCAGTCAAGG |
| *NtCUC4* | CACCAGAAGAGTGCAGGAATGAAGA | CACAATTATCCAACATAGAAGAAG |
| *NtFHY3* | CACCCATGGTGAAGCTTATGCC | GGAATTAAGACTTTGCATCTGAATG |
| *NtLOM1* | CACCAGCTATTCAAAGCTGCAG | AACTTTCTCTAGTGAGTCCAAGCTC |
| *NtLOF1* | CACCAGTAGTAGTAATATTGGCAAGGAA | CTTTACTACTACTGACAAATAAGCTT |
| *EA1* | CACCACAAGAAATGTTCCAATAAAGAT | TTGCTTGATTCCTCGTTTCCCTT |
| *EA2* | CACCTCACTCAGAGAGATGAAGATGA | TGAAATTTGTGATTCTGCATA |
| *EA3* | CACCAATCTGTCGGAGAGTTGCAC | GTACGGAGTCTAGCACATAATTGGA |
| *EA4* | CACCTGAAATGAGGCAGAAACTTATG | TAACCAATGTAGCCTGATCAACTT |
| *EA5* | CACCACTTTGTCGAATGCCGGAAT | TCTGGATTGAAGTATTTGGCAACT |
| *EA6* | CACCGGAAATGAGACAGAAGCTAGTG | ACCAATGTGGCCTGATCCAAAG |
| *EA7* | CACCGTGATATCCATGAAATCTTATCTT | TTATTGGAGCTGCATGGGAT |
| *EA8* | CACCACAATGTCAAGCAGAAGATCAC | GTTGCTATCATCACATAAGTAAGC |
| *EA9* | CACCAACCATGAGCAAGAGATTAAGAG | AATCCTTCAGTTTTCGGAGCTTTA |
| *EA10* | CACCTTTAGGGTTATGAATATGAGGG | GTCATAAAGCATTCCTTTGAA |
| *EA11* | CACCACTGATAATTTTGGTCAAAGAAC | TTCGAAATTGGTCACTGCATT |
| *VE1* | CACCACAAATAGTAGCGGAAGC | TGCTGCAATTACCATTACTGGA |
| *VE2* | CACCAGACATCAAAAGAGGTCCCTTTA | GAATTTCCTAAATGCTTCTCCTATC |
| *VE3* | CACCGTGGTGCATGCAAATATCTTAG | CTCCAGTTCTTCATCTAACACAT |
| *VE4* | CACCTGGTTCGAAAAACAAGCCAAA | GGCAATCTCTCATAAGCAACAT |
| *VE5* | CACCCTTGTGGTGCATGCAAGTTTCT | GCTTGAAGTAGTGCAGAAGATATC |
| *VE6* | CACCCACAAGACTCAACAAGGGACAG | CATAAGCTCCATGACCTTGAC |
| *VE7* | CACCTTCTTCAAGCAAAATTAATGAC | ATTAGAGTCATGAGCCATTAGC |
| *VE8* | CACCTTTTGTTGAAAGTTGTTCTATAA | GGAGAACACAAGACCTGAACCT |
| *VE9* | CACCTGAAGGAAGCGAGACTCTGC | GCCCTCCTGTGCACTCTTGAC |
| *VE10* | CACCGGGGTCCAAGAACAAGG | CTCTAATGGCAATCTCTCATATGC |
| *VE11* | CACCGGTTTTAGGTTCCATCCAACTG | CCCTTGAAATTACCCACTCGTCC |
| *VE12* | CACCACCTGGTTTTAGGTTTCATCC | GTATTCTGCATATCACCCATTCC |
| *VE13* | CACCGAGCTTGCTCAACTCCAAG | TAGCTGGTTTCTCAATTTCTCGAC |

Supplemental Table S3. Labor hours for manually removing lateral shoots in field trials at commercial production sites.

| Location | Line | Labor hours relative to Coker319 (%) |
| --- | --- | --- |
| 1 | Coker319-*ls*-*1* | 57.9 |
| 2 | Coker319-*ls*-*1* | 58.4 |
| 3 | Coker319-*ls*-*1* | 25.0 |
| 4 | Coker319-*ls*-*1* | 52.3 |

The location numbers are the same as those in Table 5. At all locations, chemical “suckercides” were applied according to standard cultivation practices. Emerging lateral shoots were manually removed.

Supplemental Table S4. Accession numbers of genes screened by RNA-seq

| Gene | Accession numbers | |
| --- | --- | --- |
|  | S-gene | T-gene |
| *VE1* | LC613172 | *Nitab4.5_0000647g0170.1* |
| *VE2* | LC613173 | LC613174 |
| *VE3* | LC613175 | *XM_016659109.1* |
| *VE4* | LC613176 | *XM_016639435.1* |
| *VE5* | *XM_016613970.1* | LC613177 |
| *VE6* | LC613178 | LC613179 |
| *VE7* | LC613180 | LC613181 |
| *VE8* | LC613182 | LC613183 |
| *VE9* | LC613184 | *XM_016641921.1* |
| *VE10* | LC613185 | LC613186 |
| *VE11* | LC613187 | LC613188 |
| *VE12* | LC613189 | LC613190 |
| *VE13* | *XM_016590684.1* | LC613191 |
| *EA1* | LC613192 | LC613193 |
| *EA2* | *XM_016623969.1* | LC613194 |
| *EA3* | LC613195 | *XM_016586323.1* |
| *EA4* | *XM_016627120.1* | LC613196 |
| *EA5* | LC613197 | LC613198 |
| *EA6* | *XM_016658814.1* | LC613199 |
| *EA7* | LC613200 | LC613201 |
| *EA8* | LC613202 | LC613203 |
| *EA9* | LC613204 | LC613205 |
| *EA10* | LC613206 | LC613207 |
| *EA11* | *XM_016642202.1* | LC613208 |

The nucleotide sequence of each gene was obtained as mentioned in the Methods section.

The sequence information is available from Solanaceae Genomics Network (https://solgenomics.net/), GenBank (https://www.ncbi.nlm.nih.gov/genbank/) and DNA DataBank of Japan (https://www.ddbj.nig.ac.jp/index-e.html).

Accession numbers of candidate genes identified by a BLAST analysis are italicized.

Supplemental Table S5. Primers and probes for quantitative PCR

| Gene | Forward primer (5′-3′) | Reverse primer (5′-3′) | Probe (5′FAM-3′TAMUA) |
| --- | --- | --- | --- |
| *NtLs* | CCGGTACTGGAAATGACCTTGA | ATCTAAGGCCTAAAGAGTGAGCAAAT | CCCTTCGTAGAACCGGAGATCGTTTAGCT |
| *NtBl1* | GAGAAAACAAATGTAAGTACACCATTAGG | GAAAAAGTTTGAATCTTCTTGCCAA | GATTTGAAAGGGCGTTTGGGTATGGG |
| *NtBl2* | AGCCATGTCAAGATCAGAGCTTAA | TGTCTTTTCCATAGCTCATTTGGTT | AATCCCATGCAAGCAACTTGTTCCTCA |
| *NtBl3* | AATGAAGATGATCAGAGTAATTCCAAGT | TGGATTTTGTTGTTCAACATAACAACT | ATTGAAGGTGGAGGAAAGCCAATTAATGGA |
| *NtREV* | TCTCCAGGCTCCCCTGAAG | TGTCCCCATGTGATAACTGTAGCT | AACGTTGTCGCACTGGATCTGCCA |
| *NtCUC1* | GTAGGGACTTTAGCTTTAGCGATTG | TGGAGCCAAATCTATACACAAACAG | TCAATTTCTGCCCCCTGTCCCACATTCA |
| *NtCUC2* | CTGGACTTACTGAAATTCTTGACAG | TGTTGAATGCAACTTAGGGGATTA | TGAAGCTCTACTCATCTCTCTGCTTTTCCT |
| *NtCUC3* | GATGAAAATGAAATGGCGAATAGTAA | GCAGAAGGCTTGATCTTGCTAGT | TGTCTGCCACCATTGACAGATTCTTCCC |
| *NtCUC4* | GGGCAGAAATGAATAGTGCACAT | TTTGACAACTCCTCTTCCTTATTGATC | GTGCTGATTTGGACAACTATTGGGCT |
| *NtFHY3* | GTCCAGAGATATAATGAGCTATGCTA | TGCACGAAGAGCAAAACTATAGC | TGACCCTTCTTCACTCAATTTCATCGCCC |
| *NtLOM1* | CTACCATTTCCAAACCATGTAATTCAA | CTCTCAATTCTTGGTTGGAGCA | CTCAAACCTTCTTGAGTCGTTAGATGCCGT |
| *NtLOF1* | TGGCAGTTTGAAAATTAATAATGATGG | GGACATTGGTTTTAGTGCTAGGA | TTGTTTATGGACCAAATGGTTCACCCCAC |
| *EA1* | TGCAGTTGGAATTTCCCATGATC | GCCGAGTAGCACAAATTGAAGAA | TTTCCTTGCCCTGCACAGCTACACTTCC |
| *EA2* | GCCTAAGAAGTCAACTACCCAATTC | AGGCTACATAGCAAAATACACCAGA | CCCTCCATCAGCAGATGAAGGCTTGTCC |
| *EA3* | GCAGATGATGAGCAAATTGTTCCA | CACCTTGGGGACAGATGAAGTC | AACAGCCAACTCCGTGTTCCAGCGAA |
| *EA4* | GTCTGTTATTGAAGCAGGACCAC | ACCACATGACATCTCAAAATAAGGC | TTCAGGGCAACTTCCTCAGTGGCGAAATC |
| *EA5* | ACTCATCTACCCAGTCAACAAATCA | TGTGCTTTTCTGCAAAAGTTTAGTA | TCTACGCCGTCCTTTCCTGTTGAAGATGGC |
| *EA6* | GCCACAGAAAGGGAAACTGTTG | ACGCCATTCAGGAAGTGATCC | AGTCTGTTCTTGAAGCAGGACCACCTCT |
| *EA7* | CTTCCTTTCTCCTTCCGGTTTAG | AGGTGAAAGAACATCTTCAAGAACC | AGTGAACCGGCTCTTCAAACATCATGACAA |
| *EA8* | CTCCAAGGGGTTTTCTCTCGT | GCTAGTCTAAGCAAAGCGCG | TTTTCCCATCCCATGTGTACCTGGGTCA |
| *EA9* | ACGGTTGAGAAAGCAAAATGAAGA | CCATCGGAGATAGACTAGTTCCTC | TCATTGCACCGATCTGCTTGAAGCCG |
| *EA10* | AGGAGGTGCAGGAGAGAGC | GGATATATGGAAGCTGGATCAAACA | CCACAGCCGCCGCAAGTAGCCAAC |
| *EA11* | AGCGTACTCTGCAAATTCCTTTAG | TCAGCCAATGCTAACACTTAAGC | AACCTCGTTGCCGTCATGTATTGGTTCT |
| *VE1* | GCCTCCTCCATCCTATTAATCGA | GCGAATCCTACAAGATCCTTTCC | AGTCAGTTACCATTTAGTGGACCTCTCCGT |
| *VE2* | GCTTTTTGAGTGACAGTTCCTTG | GACAATTTAGGCTTCCACGACT | TGCTCCTTAGCAGACCATAGAGTTGGCTT |
| *VE3* | ATTCTGTTGCTGTAGATTTCGTCTC | ACACAATTAGCATTGATTAGAGAAGAA | TCTTCGTCATGGCAATTGAAGACCTGAACC |
| *VE4* | GAGTCAATTCAGATGCAACAAGGA | GGCAAATTAAGAAAAGGAAGTCCAA | ACCACTTCCACTTCCACCAGATGGTTGT |
| *VE5* | CAGGTTTAAGTGATTCGTTCGATGA | AGGTCTAAATCTGACTCCAGGTAAG | ACCAACTCTCGTGCTAATGTATGGAGCTCA |
| *VE6* | GCAGCATCTGAAGTTGAACAAGA | GCTGCACAAAATAGCTAGGGAAA | ACCGTGAAAATCCTAGCTCCGTCACTGA |
| *VE7* | ACTCCTGTTGAGAATGCACAAATAA | CCAGAAATATTAGTTTCTTCTCCTTGG | CCATCTGAAAATGCATAACCTGGAAGCTGC |
| *VE8* | TGCAGCTAGCTAAGGAAAACCC | TCTGCCCCTCTCTCCTTTTACA | CAGCGTTTGAAAAACCTGGTAGCTGCCC |
| *VE9* | AGAGCGATTCAAGAATAGATGGAAA | ATGCCACCCTTCAGTCACC | AATGTTGGGTCGATCCCTTTCAGCAGCC |
| *VE10* | CTGACCCTTCATCTGGATTTCC | GTACTGCAAACTATACGTCACGT | CAGACTCCAAATGGTAGCTGACCATTAGGC |
| *VE11* | GCCCACTACACAAGGAACTCAA | TGGAGCAGGAAAACCGCTG | TCACTCCATGAGAATCTGCAGCTTCCGTTA |
| *VE12* | ATGGCTACCCTACAAGCTTGAAA | TTGCCAATGTGTAGTTGTTGTGG | TCTTAACACAGCAACATCAGCAGAAGCAGC |
| *VE13* | GCTTGCAAATCCTTATCATTGGAA | GGCTTACCTCAAGTTTTAGATTACTG | CATTAACTCCGTCTGCAGTAACTTGAGCGG |

Supplemental Table S6. Primers for screening mutations

| Gene | Forward primer (5′-3′) | Reverse primer (5′-3′) |
| --- | --- | --- |
| *NtLs-S* | CTTGACACCATCTAATGTTGTTG | ATCTAAGGCCTAAAGAGTGAGCAAAT |
| *NtLs-T* | GTGGAGGCTTTGGATTATTATG | GAAGACCTCTTTGTCCTTCACCATGCAG |
| *NtLs-T* | ACACCTAATGCATCATCTAATGTT | ATCTAAGGCCTAAAGAGTGAGCAAAT |
| *NtBl1-S* | CTTTTTGTGGAAATTTTTTCTCTAGT | CTTGATAGTAGTAGTACCAAAATACAC |
| *NtBl1-T* | GAAAACTTTTCCTTTTGTAAACCA | CTTGAGTAGTAGTAGTAGTATTTCCAAA |
| *NtREV-S* | TTTGGAATTGAGGGTGAACATTGTGC | ACGTTACCATTCGTCTACAGTAAGC |
| *NtREV-T* | TCGATTGGGTTGTATGAGTTAACCGT | GTTACCATAAGCTGTGGAATATCAGG |
| *VE7-S* | GCATGGACAATCTCATCTTCTC | CTGGGCAATATTCCACCATT |
| *VE7-S* | AATGGTGGAATATTGCCCAG | ACAACATACCATACTACCACACACTA |
| *VE7-T* | TGCATGGACAATCTCCTCTT | CAACAGGAGTTGAGTTATTCTCAT |
| *VE7-T* | ATGAGAATAACTCAACTCCTGTTG | ACAACATACCATACTACCACACACTA |
| *VE12-S* | GTGTACCAGCTAGTTATTATTGCG | CCTGATCCGTTCTGATAGATCG |
| *VE12-S* | ATTTGTTAAAAAGTTGTAATAAAATTGG | TTTCTTTGAATTGCTAACGAGGA |
| *VE12-T* | TGCATTAACATGAATGCGAC | TCTAAATAGCGAGTAATAAGGATGAGA |
| *VE12-T* | GTTTGTTAAAAAATTGTAATAAACTTGG | TTTCTTTGAAGTGCAAAAGGAAT |

Supplemental Table S7. CIELAB color scores (International Commission on Illumination; http://cie.co.at/) determined using Leaf Color Chart 2019A for tobacco

| Color score | 1 | 2 | 3 | 4 | 5 | 6 | 7 | 8 | 9 | 10 |
| --- | --- | --- | --- | --- | --- | --- | --- | --- | --- | --- |
| L* (lightness) | 86 | 80 | 74 | 68 | 62 | 55 | 45 | 42 | 39 | 35 |
| a* (red/green value) | -1 | -5 | -10 | -15 | -17 | -17 | -16 | -15 | -15 | -13 |
| b* (blue/yellow value) | 31 | 47 | 46 | 44 | 37 | 28 | 21 | 17 | 14 | 9 |
