# Supplementary material for: Inhibition of lateral shoot formation by RNA interference and chemically induced mutations to genes expressed in the axillary meristem of Nicotiana tabacum L
Source: BMC Plant Biol. 2021 May 27;21:236. doi: 10.1186/s12870-021-03008-3 (PMC8157709; doi:10.1186/s12870-021-03008-3)
Supplement: Supplementary file 2 — Additional file 2: Supplemental Fig. S1. Seed yield of mutant lines. Statistical significance was determined using the t-test (**p < 0.01). NS: not significant. [file 12870_2021_3008_MOESM2_ESM.pptx]

## Slide 1
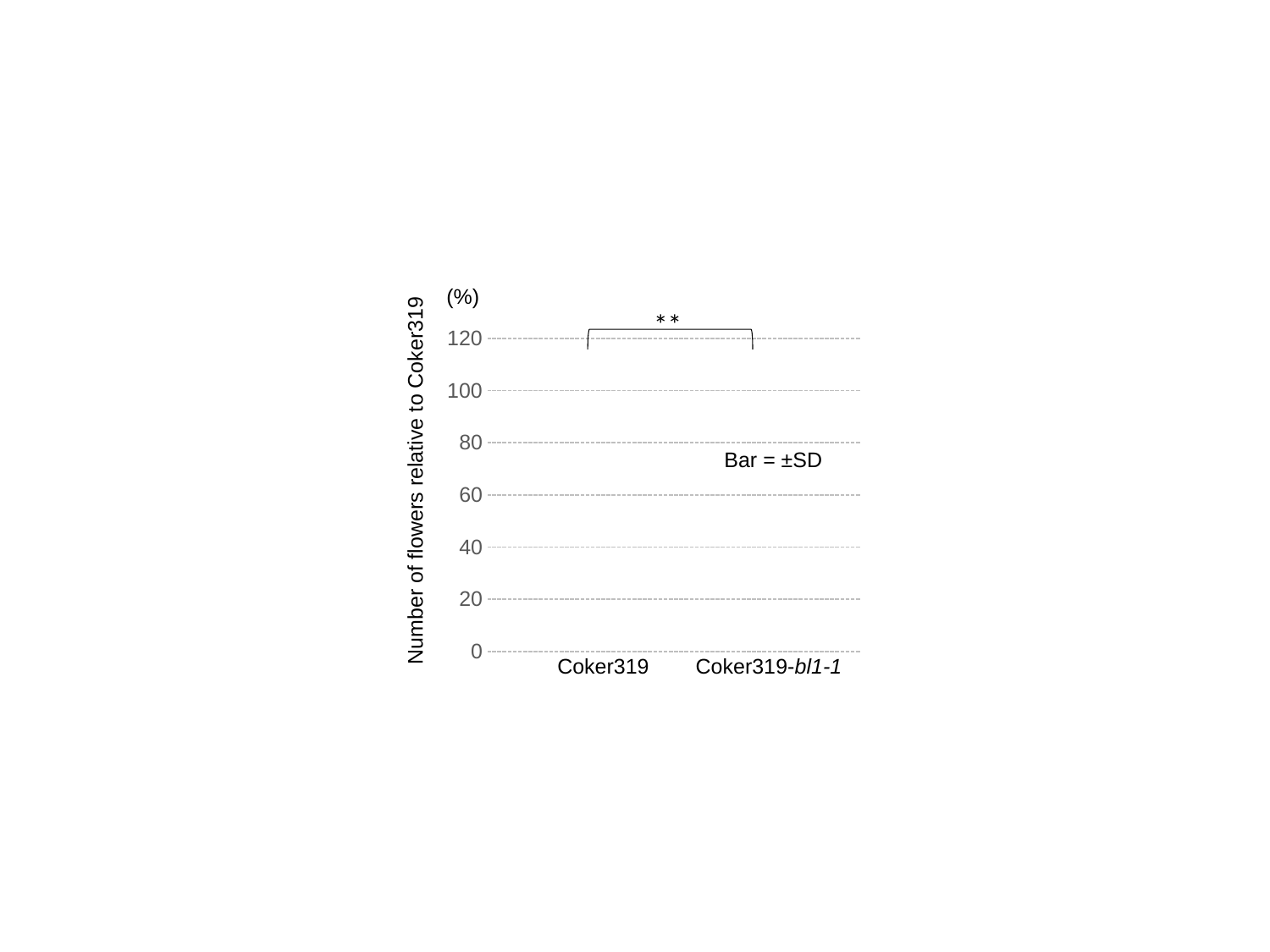

(%)
Number of flowers relative to Coker319
**
### Chart
| Category | 系列 1 | 系列 2 |
|---|---|---|
| ME | None | 100.0 |
| IE | None | 26.835664335664337 |Bar = ±SD
Coker319
Coker319-bl1-1
